# Supplementary material for: Planar Boronic Graphene and Nitrogenized Graphene Heterostructure for Protein Stretch and Confinement
Source: Biomolecules. 2021 Nov 24;11(12):1756. doi: 10.3390/biom11121756 (PMC8698321; doi:10.3390/biom11121756)
Supplement: Supplementary file 1 [file biomolecules-11-01756-s001.zip › biomolecules-1462148-supplementary.pdf]

Supporting Information For

Planar Boronic Graphene and Nitrogenized Graphene Heterostructure for Protein Stretch and Confinement

Xuchang Su[1,2], Zhi He[2], Lijun Meng[2], Hong Liang[1], and Ruhong Zhou[2,3*]

[1] Department of Physics, Hangzhou Dianzi University, Hangzhou 310018, China

[2] Institute of Quantitative Biology, College of Life Sciences, Zhejiang University, Hangzhou 310027, China

[3] Department of Chemistry, Columbia University, New York, NY 10027, USA

* Correspondence should be addressed to rhzhou@zju.edu.cn

Context:

Fig. S1 Analysis of end-to-end distances for polyglutamine (polyQ_{42}) in solution, on boronic graphene (BC_3), and the nitrogenized graphene (C_3N) stripe.

Fig. S2 Analysis of end-to-end distances for α-Synuclein (α-$Syn_{61–95}$) in solution, on BC_3, and the C_3N stripe.

Fig. S3 Detailed analysis of the polyQ_{42} stretching process on the BC_3/C_3N/BC_3 heterostructure.

Fig. S4 Detailed analysis of the α-Syn_{61-95} stretching process on the BC_3/C_3N/BC_3 heterostructure.

Fig. S5 Influence of the C_3N stripe width on the potential well for the straightened Aβ peptide.

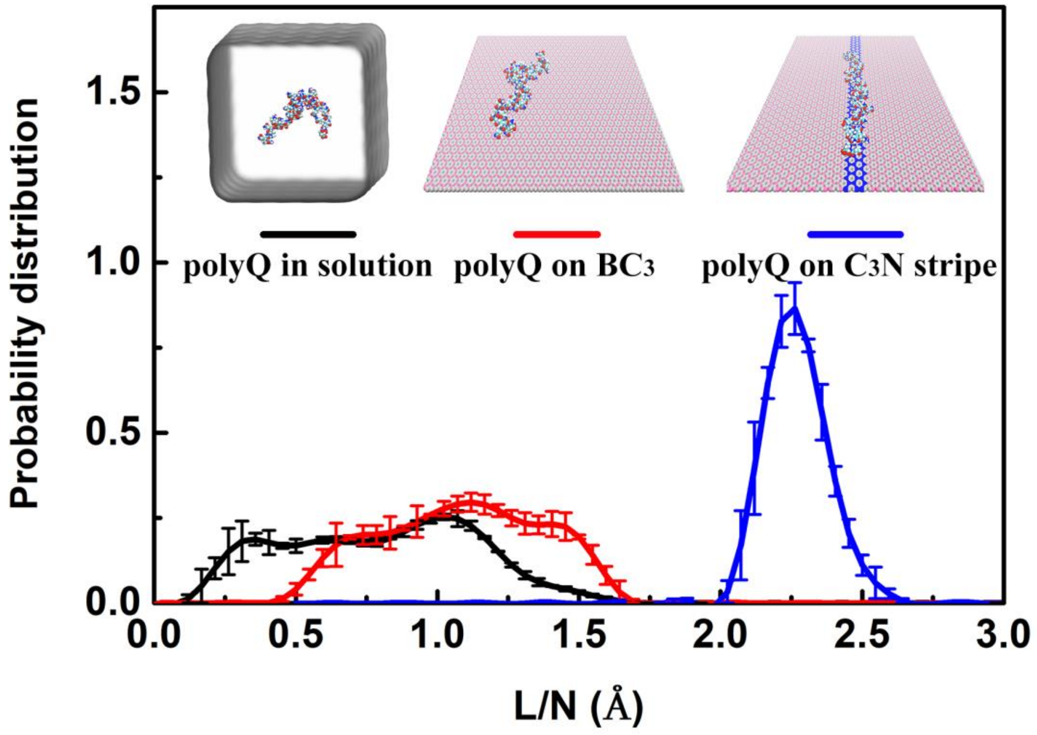

Fig. S1 Probability distribution of end-to-end distances for polyQ in solution (black line), on BC_3 (red line), and the C_3N stripe (blue line).

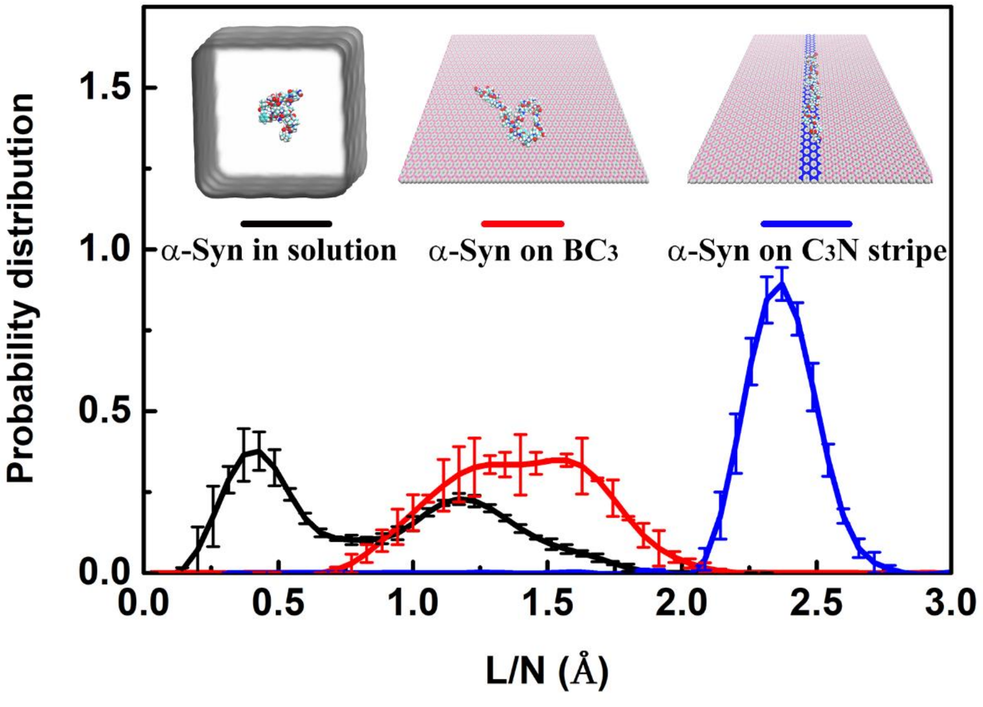

Fig. S2 Probability distribution of end-to-end distances for α-Syn in solution (black line), on BC_3 (red line), and the C_3N stripe (blue line).

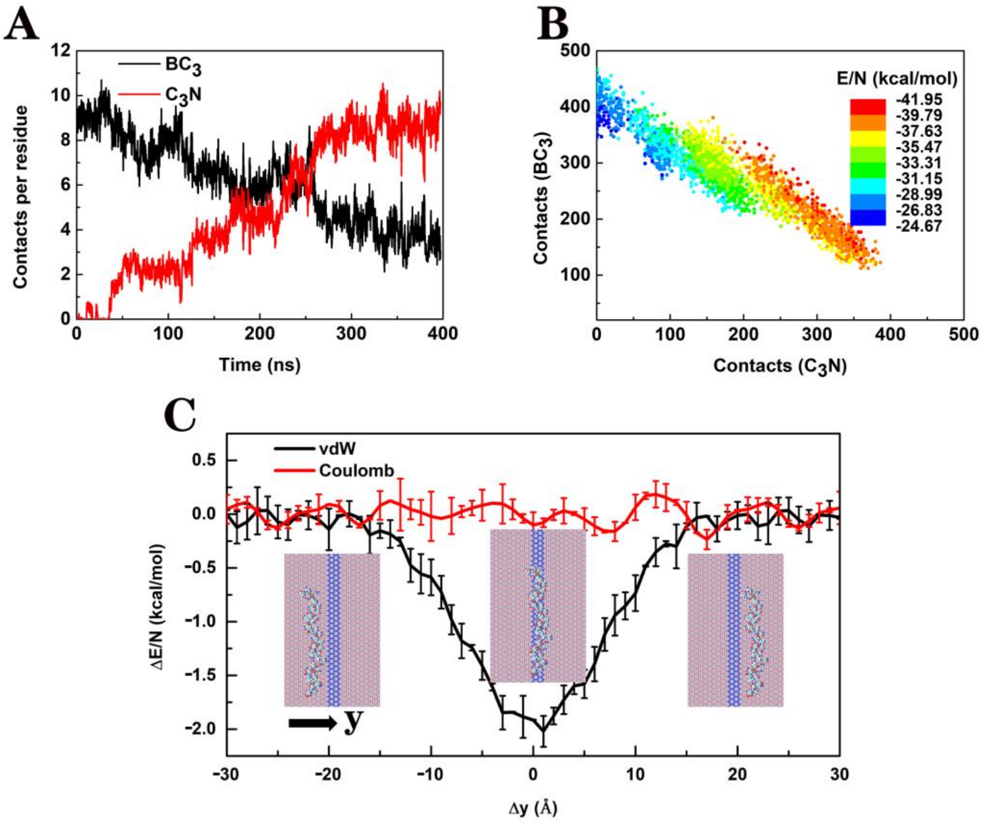

Fig. S3 (A) Number of atoms in $BC_3/C_3N/BC_3$ that are within 4.0 Å of the polyQ peptide throughout the simulation. (B) A scatter plot of the number of atoms in $BC_3/C_3N/BC_3$ in contact with polyQ. The color represents the average interaction energy per residue between polyQ and $BC_3/C_3N/BC_3$. (C) A smoothed curve of van der Waals (black line) and Coulomb (red line) interaction energy between polyQ and $BC_3/C_3N/BC_3$, when the elongated polyQ peptide moved across the C_3N stripe in the y-direction.

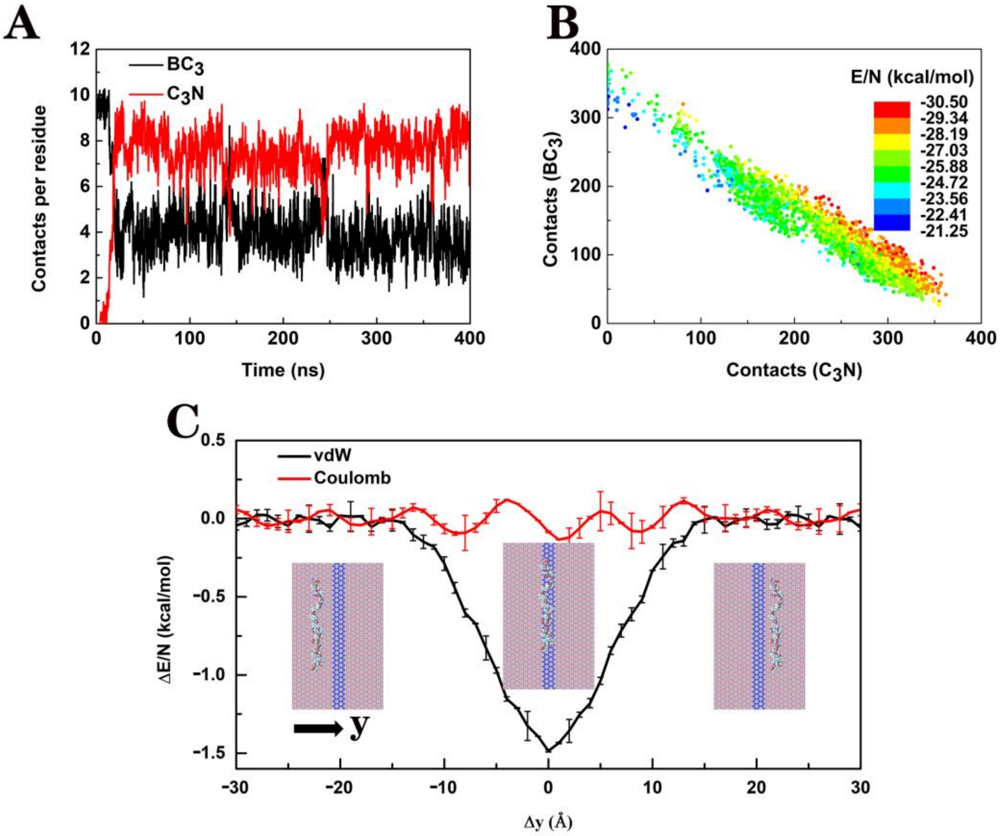

Fig. S4 (A) Number of atoms in BC_3/C_3N/BC_3 that are within 4.0 Å of the α-Syn peptide throughout the simulation. (B) A scatter plot of the number of atoms in BC_3/C_3N/BC_3 in contact with α-Syn. The color represents the average interaction energy per residue between α-Syn and BC_3/C_3N/BC_3. (C) A smoothed curve of van der Waals (black line) and Coulomb (red line) interaction energy between α-Syn and BC_3/C_3N/BC_3, when the elongated α-Syn peptide moved across the C_3N stripe in the y-direction.

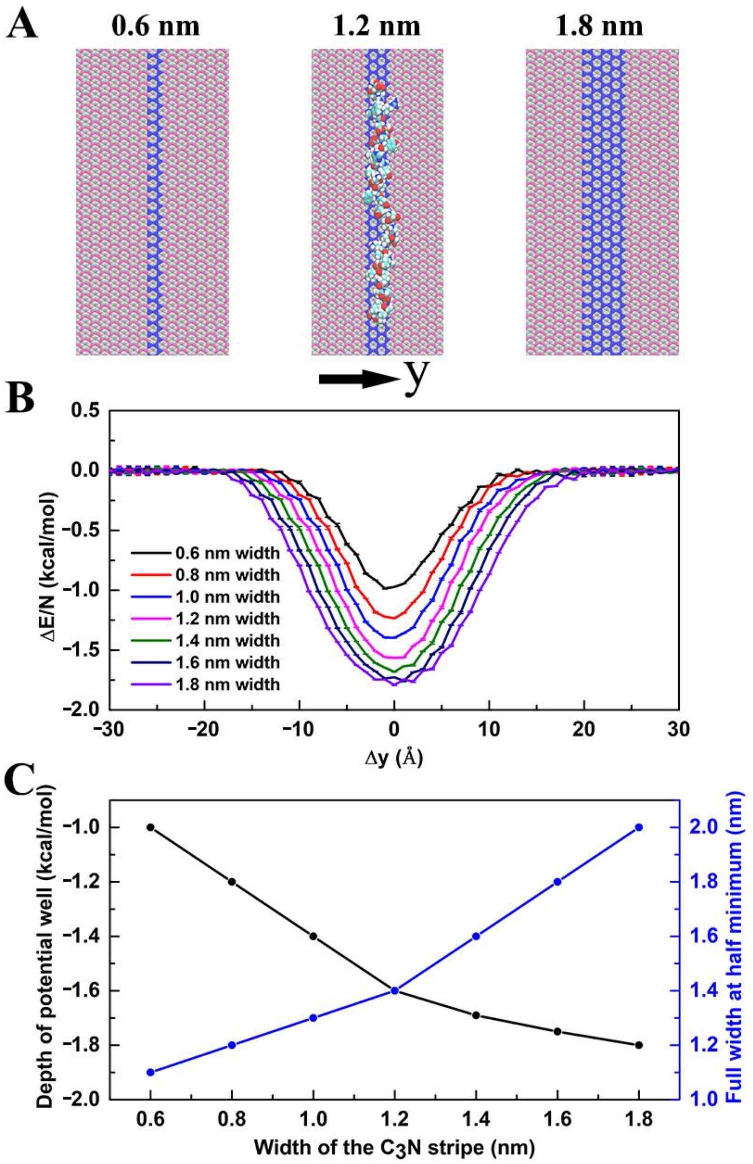

Fig. S5 Influence of the C_3N stripe width on the potential well for the straightened Aβ peptide. (A) Representative illustrations of C_3N stripes with widths of 0.6 nm, 1.2 nm, and 1.8 nm, respectively. The Aβ peptide elongated on the 1.2-nm-width C_3N stripe was used for the energy scan of each potential well. (B) Smoothed curves of van der Waals interaction energies between Aβ and $BC_3/C_3N/BC_3$, when the elongated Aβ peptide moved across each C_3N stripe with different widths in the y-direction. (C) The "potential-depth" (black line) and the "potential-width" at half-minimum of each well as a function of the C_3N stripe width.
